# Adsorptive Removal of Methylene Blue from Aquatic Environments Using Thiourea-Modified Poly(Acrylonitrile-*co*-Acrylic Acid)

**DOI:** 10.3390/ma12111734

**Published:** 2019-05-28

**Authors:** Abel Adekanmi Adeyi, Siti Nurul Ain Md Jamil, Luqman Chuah Abdullah, Thomas Shean Yaw Choong, Kia Li Lau, Mohammad Abdullah

**Affiliations:** 1 Department of Chemical and Environmental Engineering, Faculty of Engineering, Universiti Putra Malaysia, UPM Serdang 43400, Malaysia; abeladeyi@abuad.edu.ng (A.A.A.); chuah@upm.edu.my (L.C.A.); csthomas@upm.edu.my (T.S.Y.C.); laukiali@hotmail.com (K.L.L.); 2 Department of Chemical and Petroleum Engineering, College of Engineering, Afe Babalola University Ado-Ekiti, ABUAD, KM. 8.5, Afe Babalola Way, P.M.B. 5454, Ado-Ekiti, Ekiti State, Nigeria; 3 Department of Chemistry, Faculty of Science, Universiti Putra Malaysia, UPM Serdang 43400, Malaysia; 4 Centre of Foundation Studies for Agricultural Science, Universiti Putra Malaysia, UPM Serdang 43400, Malaysia; 5 Institute of Tropical Forestry and Forest Products (INTROP), Universiti Putra Malaysia, UPM Serdang 43400, Malaysia; 6 Faculty of Chemical Engineering, Universiti Teknologi Mara, Masai 81750, Johor darul Takzim, Malaysia; moham3767@johor.uitm.edu.my

**Keywords:** adsorption, kinetics, redox polymerization, thioamide groups, regeneration

## Abstract

The paper evaluates the adsorptive potential of thiourea-modified poly(acrylonitrile-*co*-acrylic acid), (TA-poly(AN-*co*-AA)) for the uptake of cationic methylene blue (MB) from aquatic environments *via* a batch system. TA-poly(AN-*co*-AA) polymer was synthesized through redox polymerization and modified with thiourea (TA) where thioamide groups were introduced to the surface. Fourier transform infrared (FT-IR) spectroscopy, scanning electron microscopy (SEM), CHNS and Zetasizer were used to characterize the physico-chemical and morphological properties of prepared TA-poly(AN-*co*-AA). Afterwards, it was confirmed that incorporation of thioamide groups was successful. The adsorption kinetics and equilibrium adsorption data were best described, respectively, by a pseudo-second-order model and Freundlich model. Thermodynamic analysis showed the exothermic and spontaneous nature of MB uptake by TA-poly(AN-*co*-AA). The developed TA-poly(AN-*co*-AA) polymer demonstrated efficient separation of MB dye from the aqueous solution and maintained maximum adsorption capacity after five regeneration cycles. The findings of this study suggested that synthesized TA-poly(AN-*co*-AA) can be applied successfully to remove cationic dyes from aquatic environments.

## 1. Introduction

Water is usually critical for human life and for the survival of almost all ecosystems. It is also a critical feedstock in a variety of key industries such as food, pharmaceuticals, agriculture, electronics, printing, paper, leather, plastics and cosmetics. These industries have been found responsible for the escalating release of coloured effluents contains synthetic organic dyes. The discharge of coloured wastewaters into the environment has adverse effects on the receiving water bodies, and inflicts noticeable hazards because they are not biodegradable and pollute natural habitat [1,2,3,4,5]. Synthetic dyes have also been reported as mutagenic and carcinogenic to both aquatic organisms and human life, causing critical heart, liver, kidney, reproductive and nervous malfunctioning [6,7,8]. Elimination of dye components from industrial wastewater to meet strict discharge regulations is, therefore, a key research area.

Techniques recently used in purification of dye-bearing wastewaters include chemical precipitation/oxidation [9], biodegradation [10], photocatalytic degradation [11], electrocoagulation [12] and adsorption [13,14]. Adsorption is ranked the most efficient separation technology for dye removal due to its design simplicity, adaptability to a wide range of dyes and easy exploitation [15,16]. However, its effectiveness largely depends on the adsorbate affinity for adsorbent materials.

Numerous adsorbents have been prepared and investigated. Applications of some adsorbents are not without limitations such as low efficiency, sludge generation, high cost and long residence time. Therefore, developing novel facile adsorbents with simple preparation route, relatively inexpensive, highly efficient and regeneration ability is required. Polymeric adsorbents and their derivatives such as polyurea, polythiophenes and polyacrylonitrile (PAN) have been synthesized for wastewater treatment, removing industrial dyes, pharmaceuticals and heavy metals. Recently, many investigations involved functionalization or surface modifications of these polymers in order to enhance their effectiveness and selectivity for specific pollutants. For instance, Gupta et al. (2014) synthesized polyaniline zirconium (IV) silicophosphate for removal of dye from aqueous solution [17]. Chen and coworkers prepared poly(cyclotriphosphazene-co-4,4′-sulfonyldiphenol) nanosphere and employed as adsorbent to uptake of methylene blue [18]. Core@shell poly(acrylic acid) microgels/polyethersulfone beads synthesized by Chen et al. (2017) for adsorptive removal of dyes [19]. Surface functionalization of Fe_3_O_4_ nanoparticles with L-arginine for reactive blue 19 azo dye uptake from water was also reported by Dalvand and coworkers in 2016 [20]. Similarly, Zare et al. prepared dextrin-g-poly m-phenylenediamine (DgPmPDA) by chemical graft polymerization for removal of Pb(II) and methylene blue from wastewater [21]. 

In this study, poly(acrylonitrile-*co*-acrylic acid) copolymer (block copolymer according to Bajaj et al. (1993)) [22], was synthesized via redox polymerization and its surface was chemically modified with thioamide groups and denoted as TA-poly(AN-*co*-AA). Thiourea was selected due to the possession of a high content of nitrogen and sulphur as electron-rich and aptitude to fasten pendant polymer chain [23,24]. The structural and morphological features of the as-synthesized polymer was characterized by FTIR, SEM, CHNS, TGA, BET and zeta potential. The TA-poly(AN-*co*-AA) was used as a high capacity adsorbent for methylene blue (MB) uptake from aqueous environment. MB is selected as a linear molecular structure cationic dye compare to malachite green (MG) with triangular molecular structure reported in our previous work [25]. This is to ascertain whether molecular structure directly affects their adsorption isotherm using the same modified poly(AN-*co*-AA) adsorbent. Influence of key operation parameters such as adsorbent dose, initial concentration, pH and temperature on sorption capacity of the as-synthesized polymeric materials was explored. 

## 2. Experimental

### 2.1. Materials and Chemicals

K_2_S_2_O_8_ (potassium persulphate), NaHSO_4_ (sodium bisulphate), NaOH, HCl, CH_4_N_2_S (thiourea) (all procured from R&M Chemicals, Essex, UK), acrylonitrile (AN), acrylic acid (AA) (purchased from Acros Organics, Morris, NJ, USA); Aluminium oxide (MERCK, Darmstadt, Germany), methanol and ethanol were purchased from Systerm ChemAR (Shah Alam, Malaysia). All chemical reagents were analytical grade, used without additional purification except AN and AA. Prior to application, AA and AN was purified by passing it through AlO_3_ in a glass column. Cationic methylene blue (C_16_H_18_ClN_3_S·3H_2_O; molecular weight: 373.89 g/mol; Colour Index Number: 52015; maximum wavelength: 665 nm) was procured from the Fisher Chemicals Limited (Thermo Fisher Scientific, Winsford, UK)

### 2.2. Instrumentation

The morphologies of poly(AN-*co*-AA) and TA-poly(AN-*co*-AA) polymers were analysed with a SEM, S-3400N from Hitachi (Tokyo, Japan). Fourier transform infrared (FTIR) spectrometer, 1750X PerkinElmer Inc. (USA) and carbon hydrogen nitrogen sulphur spectrometer, CHNS-932 Leco Corporation (USA) were employed to analyse the chemical structure. Thermal profile of poly(AN-*co*-AA) and TA-poly(AN-*co*-AA) were executed using Thermogravimetric analyser (STA 6000, PerkinElmer Simultaneous Thermal Analyzer, Waltham, MA, USA). Brunauer–Emmett–Teller (BET) specific surface area were conducted by employing surface area analyser (Micromeritics Instrument Corporation, Model-3Flex, Norcross, GA, USA) and N_2_ as the adsorbing gas. Zetasizer nano series, Malvern Panalytical Limited (Malvern, UK) was used to measure the surface charge of functionalized poly(AN-*co*-AA) polymer. A UV–vis spectrometer Lambda 35PerkinElmer Life and Analytical Science (Singapore 139959, Singapore) was employed to measure the concentration of MB in a solution.

### 2.3. Synthesis of TA-poly(AN-co-AA)

**Figure 1:**
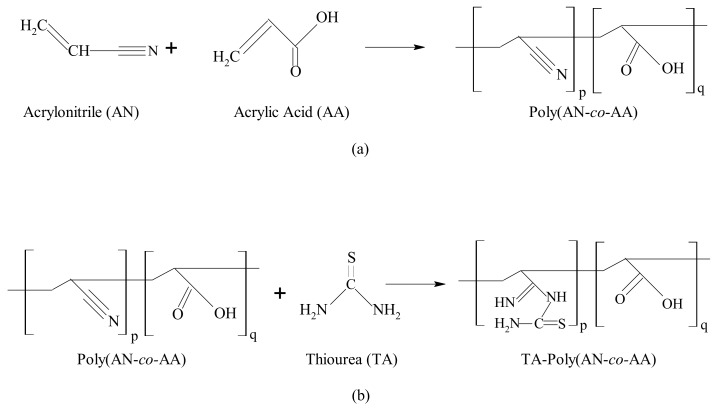
Synthesis scheme of poly(acrylonitrile-*co*-acrylic acid) (poly(AN-*co*-AA) (**a**) and its modification with thiourea (**b**).

Poly(AN-*co*-AA) were synthesized by redox polymerization as proposed in the scheme of Figure 1 as presented in our previous work [25]. Subscripts p and q are the minimal molar concentrations of AN and AA in the reactor, to maintain a 100 percent molar basis. The preparation method was similar to Zahri et al. 2015 approach, with little modification, 200 mL of double deionized water as reaction medium was first refluxed or purged for 0.5 h at 40 °C with nitrogen gas [26]. The monomers feed ratio AN/AA of 97:3 (*v*/*v*) were added to reaction medium together with initiators-sodium bisulphate (2.09 g) and potassium persulphate (2.16 g). The reaction was constantly stirred at 200 rpm for 2 h at 60 °C. The resulting polymer, poly(AN-*co*-AA) was precipitated in methanol for an hour. It was successively washed with deionized water/methanol solution, dried until constant weight in a *vacuo* at 40 °C.

The thioamide incorporation conditions of poly(AN-*co*-AA) were selected according to our previous work [25]. Briefly, 6 g of thiourea was stirred with deionized water/ethanol mixture at 200 rpm, 70 °C for 0.5 h. Thereafter, 5 g of the synthesized poly(AN-*co*-AA) was introduced to the solution and the surface functionalization reaction allowed for 5 h. The solid formed denoted as TA-poly(AN-*co*-AA) soaked in ethanol/deionized water, filtered and dried overnight at 50 °C.

### 2.4. Batch Adsorption Study

To investigate influence of adsorption process parameters, kinetics, isotherm and thermodynamics, batch adsorption experiments were performed trice to justified measurements. One variable at a time (OVAT) approach was adopted for optimization. The stock solution of MB was prepared by dissolving required mass of MB dye in deionized water. Various experimental concentration were prepared by dilution of required volume from the stock solution. Regulation of pH was done with addition of NaOH and HCl to test solution. Batch experiments were carried out in a temperature controlled waterbath shaker (Model-903, Tech-Lab Mfg Sdn Bhd, Malaysia) at 100 rpm, using 100 mL of MB solution with desired concentration and known TA-poly(AN-*co*-AA) dose. The samples were withdrawn at set time intervals (0–120 min) and change in MB concentration was measured by UV–vis spectrophotometer.

The quantity (mg/g) of MB adsorbed at equilibrium $q_{e}$ and at any time, $q_{t}$ were calculated respectively using Equations (1) and (2);

(1) $$q_{e}=\frac{(C_{o}-C_{e})V}{m}$$

(2) $$q_{t}=\frac{(C_{o}-C_{t})V}{m}$$

where,$C_{o}$, $C_{e}$ and $C_{t}$ are the liquid-phase concentrations (mg/L) of MB dye at initial, equilibrium and at any time t. Here V (L) is the solution volume and m (g) is the mass of the TA-poly(AN-*co*-AA) used.

The extent of MB removed in percentage (%Re) was calculated by using Equation (3):

(3) %Re=(Co−Ce)×100Co

Desorption studies were carried out by agitating 0.5 g of TA-poly(AN-*co*-AA) with 100 mL of 100 mg/L MB solution in 250 mL Erlenmeyer flask for 24 h to prepare saturated materials. Sample was washed with distilled water and left to dry. Then, 0.2 g of saturated sample was mixed with 40 mL of 1 M nitric acid and 0.5 M thiourea mixture (50:50) and agitated for 2 h, after which concentration of solution determined. Thereafter, the regenerated adsorbent was filtrated, washed with distilled water and dried at 50 °C in vacuum oven overnight for the next adsorption experiment. Equation (4) was used to calculate the regeneration efficiency (*R*%):

(4) $$R(\%)=\frac{adsorption\;capacity\;of\;regenerated\;polymer}{adsorption\;capacity\;of\;fresh\;polymer}\times 100$$

## 3. Results and Discussion

### 3.1. Characterization of TA Functionalized poly(AN-co-AA) Adsorbent

**Figure 2:**
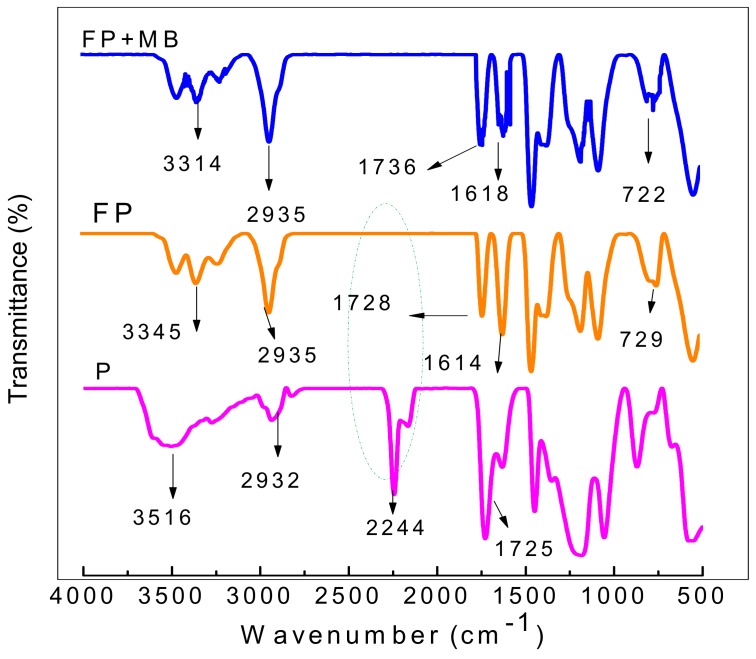
FTIR spectra of synthesized poly(AN-*co*-AA) (P), TA- poly(AN-*co*-AA) (FP) and MB-loaded TA-poly(AN-*co*-AA) (FP+MB).

Figure 2 shows the FTIR spectra of the synthesized poly(AN-*co*-AA), TA-poly(AN-*co*-AA) and evaluates post adsorption changes in chemical structures of MB-loaded TA-poly(AN-*co*-AA). The absorption band at 2244 cm^−1^ is attributed to –C≡N stretching of acrylonitrile. The peaks at 1725/1728 cm^−1^, and 2932/2935 cm^−1^ and 3516 cm^−1^ are assigned to the –C–H and –C=O bonds, respectively. Post functionalization of poly(AN-*co*-AA), the obtained spectrum (FP) shows significant changes. The band at 2244 cm^−1^ completely disappeared, and has been converted to –C=N– at 1614 cm^−1^ in TA-poly(AN-*co*-AA) polymer. The characteristic peaks of O-H and N-H functional groups overlapped around 3500–3200 cm^−1^ [26,27]. Absorption bands at 729 cm^−1^ and 1015 cm^−1^ correspond to C=S group vibrating region attached to nitrogen [27,28]. Notable changes in spectrum of poly(AN-*co*-AA) after functionalization inveterate successful incorporation of thioamide group to the polymer surface. Similar trends were reported by other researchers [27]. After dye adsorption, the corresponding spectrum shows some changes such as shifts in absorption peaks assigned to –OH, –NH_2_ and –C=S functional groups. This suggested that presence of these functional groups at the TA-poly(AN-*co*-AA) surface possibly aids entrapment of cationic MB dye from liquid phase. Similar results were reported by [29] for adsorption of chromotrope dye onto activated carbons.

In addition, the actual composition of synthesized poly(AN-*co*-AA) (P) copolymer was determined as 89.48:10.52 (AN:AA), using optical density ratio (ODR) value for the reduction of physical variations error (Supplementary Data). A similar method was reported by [30,31].

**Figure 3:**
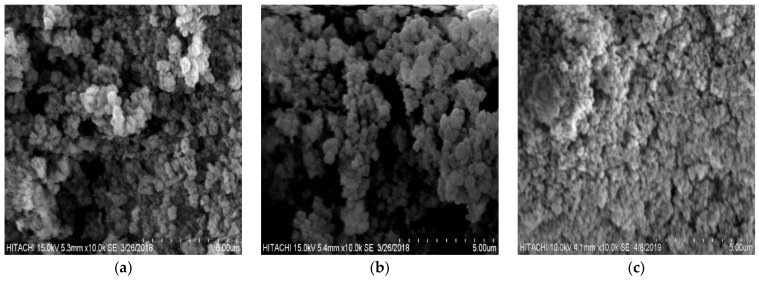
SEM micrographs of (**a**) poly(AN-*co*-AA), (**b**) TA-poly(AN-*co*-AA) and (**c**) MB-loaded TA-poly(AN-*co*-AA).

Figure 3 shows the SEM micrographs of the unmodified, modified poly(AN-*co*-AA) and MB-loaded modified poly(AN-*co*-AA) polymers. From the images both samples were spherical in shape, rough surface (suitable for adsorption) and agglomerated features. This agglomeration and spherical nature were originated from the water content (less viscous), initiator type and intraparticle bonds of the AN-AA monomers. Aguilar and coworkers reported same morphologies for synthesized acrylonitrile/ethylene glycol dimethacrylate cross linked adsorbents [32]. The measured mean particle diameter (using ImageJ™ software, 1.52a version) revealed a slight change in poly(AN-*co*-AA) beads to 308 nm from 300 nm after chemical modification. This further confirmed the successful surface functionalization by thiourea. These observations are in agreement with the findings of [33]. Figure 3c reveals the complete change in surface morphology of TA-poly(AN-*co*-AA) after MB adsorption. MB cations cover the surrounding adsorbent particles and occupied the voids, leading to MB ion monolayer formation over the TA-poly(AN-*co*-AA) surface.

CHNS micro-elemental analyses of poly(AN-*co*-AA) and TA-poly(AN-*co*-AA) are presented in Table 1. The percentage of carbon, hydrogen, nitrogen and sulphur of functionalized polymer noticeably increased, an indication that the thiourea was incorporated successfully. This increment is also buttressed by the FT-IR spectra (Figure 2); the conversion of dipolar-nitrile groups to TA-poly(AN-*co*-AA) networks, for active binding sites and selectivity in environmental applications [34,35]. Note that the C content is as expected (increased to 61.94%; after chemical modification with thiourea). This is due to the conversion of nitrile groups to thioamide moieties along the polymer chain. On the other hand, the nitrogen content was also increased (in the case of TA-poly(AN-*co*-AA)) due to the incorporation with thiourea into polymer chain, as well. Meanwhile, the sulphur content of copolymer is 2.51% due to the presence of sulphur residue from the initiators (NaHSO_4_ and K_2_S_2_O_8_). The initiators residue was still present even after washing the polymer up to three times with polar solvent and deionized water.

**Figure 4:**
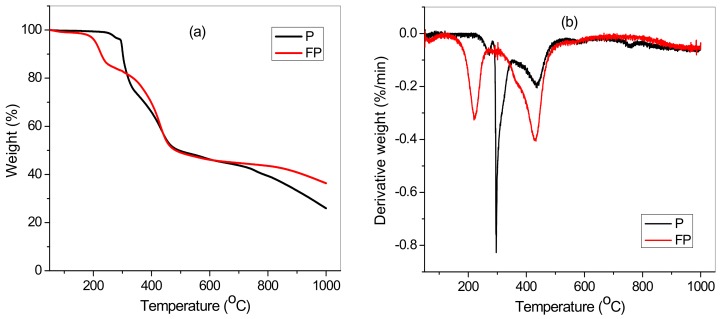
(**a**,**b**) Thermal analyses of poly(AN-*co*-AA) (P) and TA-poly(AN-*co*-AA) (FP).

Figure 4 shows the thermal analyses curve(s) for poly(AN-*co*-AA) and TA-poly(AN-*co*-AA), respectively. It revealed that TA-poly(AN-*co*-AA) decomposed faster than poly(AN-*co*-AA) between 210 and 400 °C (Figure 4a). This phenomenon is associated with the interruptions of intermolecular nitrile-to-nitrile unit arrangement of poly(AN-*co*-AA) chain due to thiourea functionalization. Besides, the intense peaks at 206 °C, 290 °C and 420 °C on the curve (Figure 4b) fell within the temperature range attributed to stabilization of polyacrylonitrile [36]. The degradation disparity exhibited by poly(AN-*co*-AA) and TA-poly(AN-*co*-AA) also corroborated the successful poly(AN-*co*-AA) modification. The result demonstrate that both samples are stable and suitable for water or wastewater treatment process [26,37].

The textural properties of poly(AN-*co*-AA) are shown in Table 2. The increment in surface area and reduction in pore volume after modification might be ascribed to attachment of thioamide group on poly(AN-*co*-AA) chains and ligand in the surface of polymer particles. As observed from Table 2, there is an increase from 41.60 nm to 47.23 nm in the pore of TA-poly(AN-*co*-AA). This is crucial to pollutant entrapment since pores act as the binding or receptor sites during adsorption process.

The as-synthesized modified polymer was negative in both acidic and alkaline conditions according to [25]. The surface charge (zeta potential) of TA-poly(AN-*co*-AA) increased negatively with increases in the solution pH from 3 to 9. Similar trends was reported by [38]. These negative surface charges proven the accessibility of functional groups at the surfaces of functionalized polymer, since thioamide group incorporation lead to hydrophobic/hydrophilic balance which enhanced adsorptive activity [23].

### 3.2. Effect TA-poly(AN-co-AA) Dose

**Figure 5:**
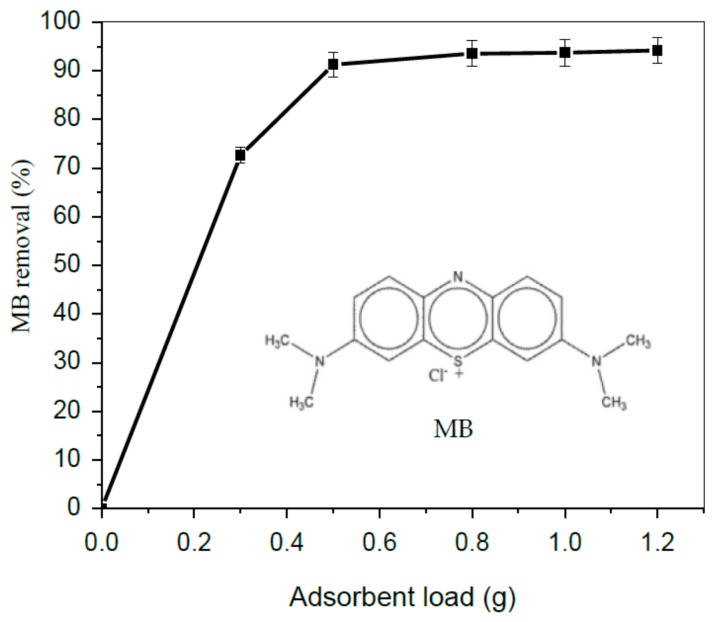
Effect of TA-poly(AN-*co*-AA) load on uptake of MB ($C_{o}$: 50 mg/L; temperature: 298 K; time: 60 min).

The quantity of adsorbent is a key factor with significant role in adsorption process. To determine optimum dose of adsorbent, mass of TA-poly(AN-*co*-AA) was varied from 0.3 to 1.2 g. Figure 5 shows the linear molecular structure of MB and the effect of adsorbent dose on adsorption. The extent of MB (in %) removal increased suddenly up to 0.5 g dosage as shown in Figure 5, this trend is attributed to an increase in area of adsorptive surface together with more binding sites availability according to [39]. Further increment of polymer dose portrayed little or no significant increase in MB % removal. Hence, 0.5 g dosage was chosen as an optimal adsorbent load for successive studies.

### 3.3. Effect of Initial pH

**Figure 6:**
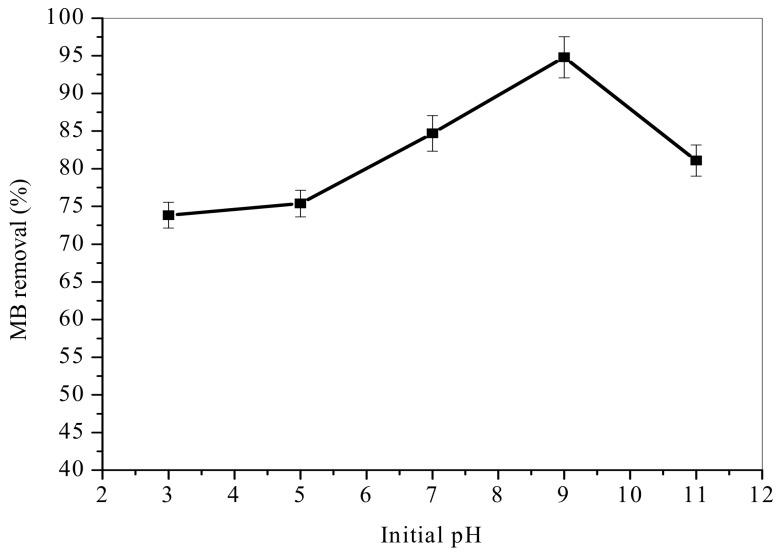
Effect of initial pH on adsorption of MB onto TA-poly(AN-*co*-AA) ($C_{o}$: 100 mg/L; adsorbent dose: 0.5 g/100 mL; temperature: 298 K; time: 60 min).

The initial pH of dye solution play key role in adsorption process and significantly affect dye solution colour and solubility [40]. The results, Figure 6 revealed a minimum MB uptake at pH 3 and a maximum at pH 9. This performance is associated to ionization of MB and adsorbent surface charge [38]. Cationic MB and anionic TA-poly(AN-*co*-AA) polymer tends to attract via electrostatic force. Results show that electrostatic interactions enhanced adsorption mechanism of organic dye on TA-poly(AN-*co*-AA) polymeric adsorbent. These results are also in agreement with zeta potential value of TA-poly(AN-*co*-AA) had highest negative surface charge at pH 9. Furthermore, π–π electron donor-acceptor can also improve the adsorption mechanism for phenolic contaminants with strong π-withdrawing potential according to Jana and co-workers [41]. H-bonding and hydrophobic/hydrophilic interaction may also contribute. However, significant dropped in adsorption efficiency was observed at pH above 9. This is probably due to hydrolysis reaction occurrence on the cyano groups of the adsorbent and less presence of negatively charged surface at pH 11. Similar reduction in adsorption capacity was reported by [42].

### 3.4. Influence of Temperature

**Figure 7:**
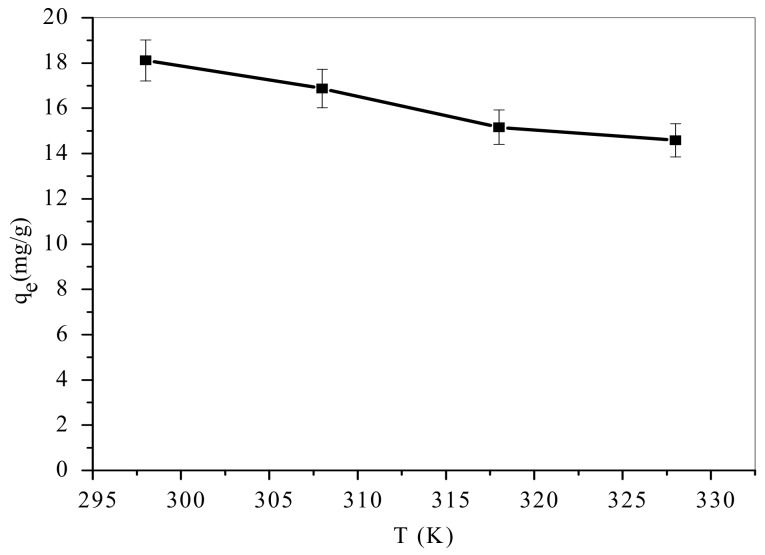
Effect of temperature on adsorption of MB onto TA-poly(AN-*co*-AA) ($C_{o}$: 100 mg/L; adsorbent dose: 0.5 g/100 mL; time: 60 min; pH: 9).

The results of temperature influence on MB uptake from aqueous solutions was examined from 298–328 K. The variation of temperature (25 °C to 55 °C) in adsorption system (Figure 7) resulted in decreased in the amount of MB adsorbed. This phenomenon is associated to increase in MB solubility and faster movement of the TA-poly(AN-*co*-AA) that made sorption sites lose contact directly with the MB ions as temperature was raised. This finding is in agreement with the report of [43,44], that the efficiency of dye adsorption is reduced at elevated temperatures. The diffusion of MB molecules to the core of TA-poly(AN-*co*-AA) requisite no extra thermal energy. Therefore, following MB sorption tests were performed at ambient temperature (25 ± 2 °C).

### 3.5. Influence of Residence Time and MB Concentration

**Figure 8:**
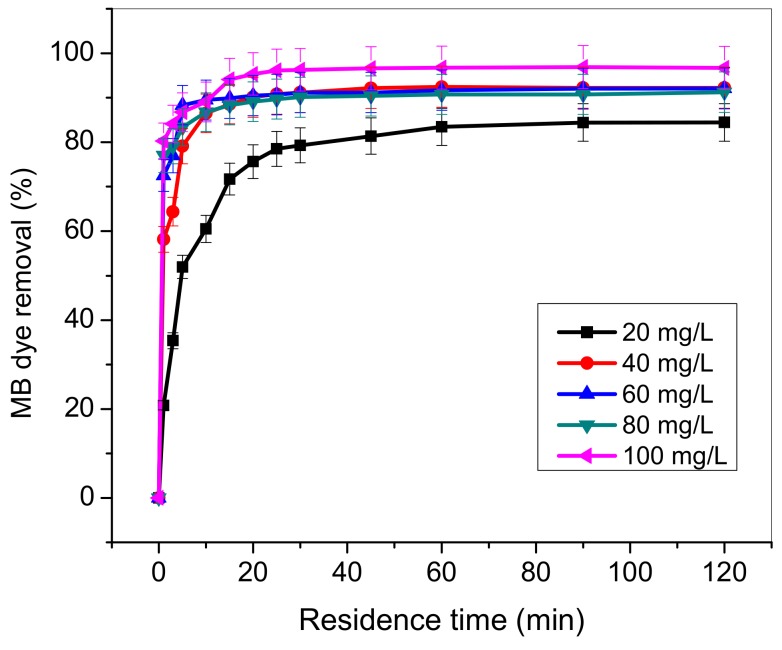
Effect of residence time on MB uptake by TA-poly(AN-*co*-AA) (initial pH: 9; adsorbent load: 0.5 g/100 mL; time; 120 min; temperature: 298 K; agitation speed: 100 rpm).

The influence of residence time on MB dye uptake by TA-poly(AN-*co*-AA) was examined for varied initial dye concentrations (20–100 mg/L). Results as presented in Figure 8 show that extent of MB removal sharply increased up to 30 min and equilibrium achieved afterwards in 1 h. Increased in MB concentrations leads to increases in amount of MB adsorbed. This finding is probably due to dynamic force propelled by MB concentration ramp to subvert the mass transfer resistance between the liquid and solid phases [45]. Equilibrium in adsorption capacity attained after 60 min caused by saturation of binding sites at high concentrations of MB. Comparable observation was report in the work of [43,46] on adsorption of dyes. Hence, subsequent studies were conducted with 60 min residence time.

### 3.6. Kinetic Studies

To comprehend the nature and rate of dye removal by TA-poly(AN-*co*-AA), pseudo-first-order (PFO), pseudo-second-order (PSO), Elovich and intraparticle diffusion (IPD) models were employed. The integral linearized form of the four kinetic models were represented by Equations (5)–(8), respectively [47,48,49,50]:

(5) $$\ln\left(q_{e}-q_{t}\right)=\ln\left(q_{e}\right)-k_{1}t$$

(6) $$\frac{t}{q_{t}}=\frac{1}{k_{2}q_{e}^{2}}+\frac{1}{q_{e}}t$$

(7) $$q_{t}=\frac{1}{\beta }\ln\left(\alpha \beta \right)+\frac{1}{\beta }\ln\left(t\right)$$

(8) $$q_{t}=k_{IP}t^{0.5}+C_{IP}$$

where, $q_{e}$, $q_{t}$ are respectively represent adsorption capacity at equilibrium and at time t, *k_1_*, *k_2_* and *k_IP_* are the rate constants of pseudo first-order, pseudo-second-order and intraparticle diffusion, intercept $C_{IP}$ reflect the boundary layer thickness effect, β and α are the Elovich constants equivalent to the extent of surface coverage and rate of adsorption at zero coverage respectively.

Table 3 showed the fitting results for the four different kinetic models. The calculated adsorption capacity $q_{e(cal)}$ from PFO model were differ significantly from experimental values $q_{e(\exp)}$. The correlation coefficients, $R^{2}$ values were not closed to 1, this implies that PFO model was not suitable to describe the adsorption mechanism of MB to the modified adsorbent. The intraparticle diffusion model did not provide a good fit according to $R^{2}$ values (Table 3), though the positive values of $C_{IP}$ indicates surface adsorption and faster initial rate [51,52].

Conversely, from Table 3 PSO kinetic model noticeably fitted well with the experimental data. The $R^{2}$ values were close to unity, indicating good agreement. In addition, the predicted $q_{e(cal)}$ values are nearer to the corresponding experimental values $q_{e(\exp)}$. Hence, the pseudo-second order model gave a best fit to the MB adsorption by TA-poly(AN-*co*-AA) for all concentration range considered. Therefore, the uptake of MB involve electron share and or transfer between cationic dye molecules and anionic polymeric adsorbent [53]. The Elovich model also provides a good fit with the experimental data based on correlation coefficient values that is greater than 0.9, which implies a multilayer adsorption. The viability of the model also confirmed by the lower values of β than α, indicate that rate of adsorption was higher than desorption [54].

### 3.7. Adsorption Isotherm

Adsorption isotherm also known as equilibrium data are fundamental to explicate and understand adsorption mechanism. Two different equilibrium models were selected to simulate the adsorption isotherms and explore the MB dye-adsorbent interactions; Langmuir and Freundlich isotherms. Non-linear and linearized form of both models are presented in Table 4 [55,56].

The important characteristics of Langmuir isotherm model expressed as dimensionless constant separation factor for equilibrium parameter, $R_{L}$ is estimated as Equation (9):

(9) $$R_{L}=\frac{1}{1+K_{L}C_{o}}$$

The $R_{L}$ values defined the type of isotherm as linear ($R_{L}=1$), favourable (0 < $R_{L}$ > 1), unfavourable ($R_{L}$ > 1) and irreversible ($R_{L}=0$) [57].

**Figure 9:**
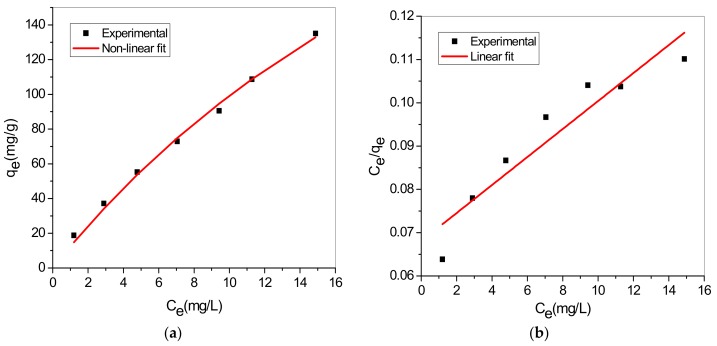
Langmuir isotherm model; (**a**) Non-linear and (**b**) Linear.

**Figure 10:**
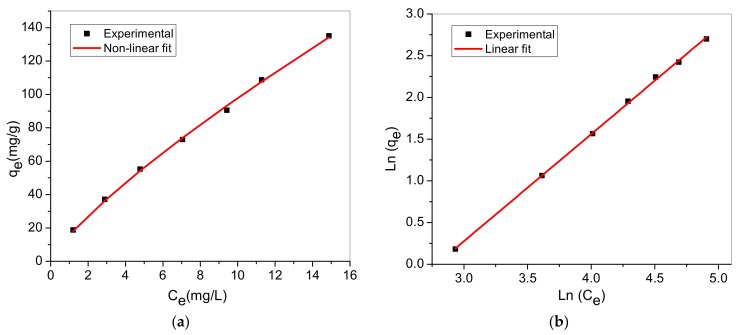
Freundlich isotherm model; (**a**) Non-linear and (**b**) Linear.

Figure 9 and Figure 10 represent model graph of Langmuir and Freundlich model, respectively. The model constants and correlation coefficient are presented in Table 5.

The results showed that the correlation coefficients ($R^{2}$) indicated that both Langmuir and Freundlich were well-fitted to the experimental data, though Freundlich model was better. Favourable MB uptake by TA-poly(AN-*co*-AA) is established because separation factor, $R_{L}$ values are fell in the range 0 and 1 (calculated values of $R_{L}$: 0.1229 to 0.5122). Also, strong bond exist between MB and TA-poly(AN-*co*-AA) adsorbent as indicated by the value of n > 1. Therefore, the adsorption process occurred at heterogeneous surfaces through multilayer adsorption system [57]. Similar adsorption isotherm was observed by [25] in the MG uptake by TA-poly(AN-*co*-AA), signify that dye molecular structure had no influence on cationic dye adsorption. Moreover, the monolayer sorption capacity of as-synthesized TA-poly(AN-*co*-AA) towards MB was high compared to other functionalized adsorbent listed in Table 6.

### 3.8. Thermodynamic Analyses

**Figure 11:**
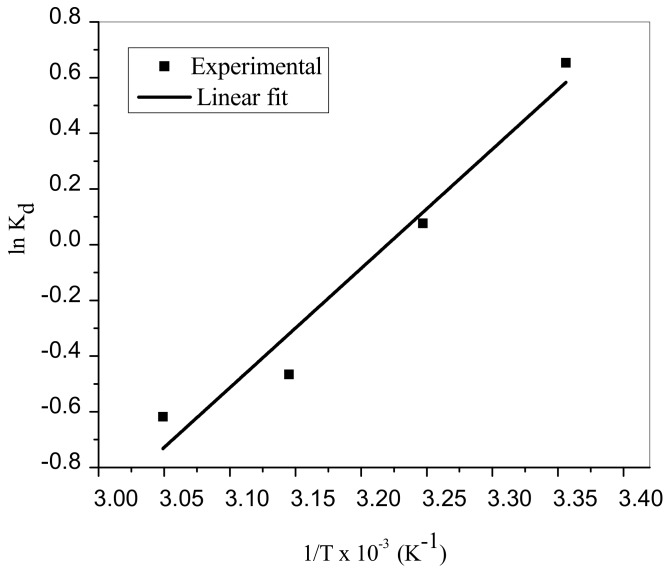
Van’t Hoff plot for MG adsorption onto TA-poly(AN-*co*-AA) (MB concentration: 100 mg/L; dosage: 0.5 g/100 mL; contact time: 60 min.; pH: 9; agitation speed: 100 rpm).

Temperature influence on MB adsorption was studied at four different temperatures 25, 35, 45 and 55 °C (Figure 7). The standard Gibbs free energy ($\Delta G^{o}$), enthalpy change ($\Delta H^{o}$) and entropy change ($\Delta S^{o}$) were deduced employing Equations (10)–(12) (Van’t Hoff thermodynamic model) [62]:

(10) $$\Delta G^{o}=\Delta H^{o}-T\Delta S^{o}$$

(11) $$\Delta G^{o}=-RT\ln K_{d}$$

(12) $$\ln K_{d}=\frac{\Delta S^{o}}{R}-\frac{\Delta H^{o}}{RT}$$

where R denotes universal gas constant, T is absolute temperature (K) and $K_{d}=q_{e}/C_{e}$ is the thermodynamic equilibrium constant. The values of thermodynamics factors $\Delta H^{o}$ and $\Delta S^{o}$ were computed from gradient and intercept of Van’t Hoff graph (Figure 11) and presented in Table 7. The adsorption process of MB is spontaneous and exothermic in nature based on $\Delta H^{o}$ value. This indicated that the energy released in bond making is higher than the energy absorbed in bond breaking between MB dye and TA-poly(AN-*co*-AA) adsorbent [56,62]. This phenomenon is further established with negative values of $\Delta G^{o}$. At higher temperature, Gibbs free energy changed from negative to positive, this suggested that desorption took place [63]. Besides, disorderliness at TA-poly(AN-*co*-AA) adsorbent-adsorbate solution interface decreases during adsorption process as indicated by the values of $\Delta S^{0}$. Similar phenomena were also reported by [64] for adsorption of MB by polyaniline nanotubes.

### 3.9. Mechanism of Adsorption

**Figure 12:**
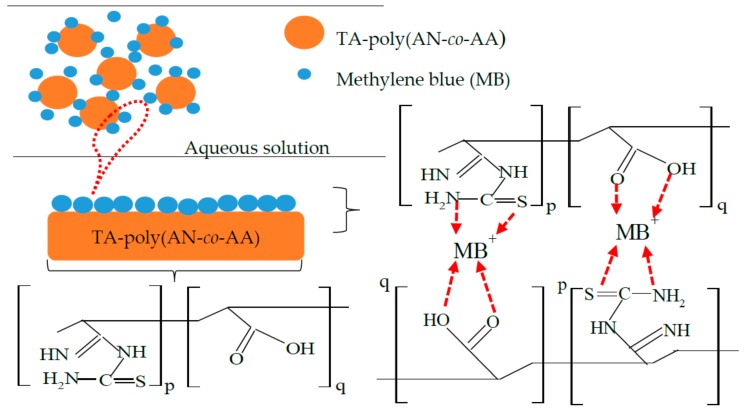
Speculated mechanism of adsorption and kinetic stages.

The mechanism of sorption of cationic MB by TA-poly(AN-*co*-AA) polymer is probably a chemisorption type as illustrated in Figure 12 due to the physicochemical properties and functional groups possessed by the adsorbent. It probably involves sharing/transfer of electrons between the adsorbate and adsorbent. The MB dye adsorption mode may involve chemical surface complexation, hydrogen bonding interactions, physical and chemisorption as suggested FTIR and SEM image of MB-loaded adsorbent. The results of zeta potential analysis show that electrostatic interactions between negatively charged TA-poly(AN-*co*-AA) and cationic MB dye molecule play crucial role in their high sorption capacities. Besides, TA-poly(AN-*co*-AA) adsorbent possess electron-rich N_2_ and S atoms which favour the adsorption process as they serve as basic reactive centre for MB uptake according to [18,27].

### 3.10. Regeneration and Reusability Study

Regeneration studies on the feasibility of desorbing MB molecules from TA-poly(AN-*co*-AA) are germane to application industrially. The technique of solvent desorption was applied by addition of nitric acid/thiourea mixture (50:50, *v*/*v*) solution to MB-loaded TA-poly(AN-*co*-AA) and shaken at 120 rpm for 3 h. The extent of MB uptake exceeds 80% after five cycles with respect to the first cycle. Thus, modified polymer demonstrate multiple used for adsorption of cationic MB dye from liquid phase.

## 4. Conclusions

In this research, a thiourea-functionalized poly(acrylonitrile-*co*-acrylic acid) polymer was synthesized by redox polymerization, and its adsorption capacity towards MB investigated via a batch system. The adsorption process of MB by TA-poly(AN-*co*-AA) was favourable at high pH, which may be attributed to electrostatic attraction between the anionic TA-poly(AN-*co*-AA) and the cationic MB dye. Equilibrium data were fitted by Langmuir and Freundlich isotherm models, signifying comprehensive adsorption with 308.64 mg/g Langmuir maximum adsorption capacity. Experimental data followed a pseudo-second-order kinetic model. Thermodynamic parameters confirmed the exothermic and spontaneous nature of the cationic MB sorption process. Reusability study conducted using mixture of nitric acid and thiourea solution as eluent revealed that prepared TA-poly(AN-*co*-AA) adsorbent can be repeatedly used for removal of dye from the liquid phase. Based on experimental findings, TA-poly(AN-*co*-AA) polymer is a promising functional regenerable adsorbent with high capacity to remove cationic dye from liquid environment. The authors are continuing to undertake follow-up on the application of TA-poly(AN-*co*-AA) in a binary and multi-component system.

## Figures and Tables

**Table 1:** CHNS micro-elemental analysis of poly(AN-*co*-AA) and TA-poly(AN-*co*-AA).

Sample/Element	C (wt.%)	H (wt.%)	N (wt.%)	S (wt.%)
Poly(AN-*co*-AA)	54.19	5.27	20.83	2.51
TA-poly(AN-*co*-AA)	61.94	5.62	25.06	3.09

**Table 2:** Textural characteristics for P and FP deduced from N_2_ adsorption.

Samples	Surface Area (m^2^/g)	Mean Pore Volume (cm^3^/g)	Average Pore Size (nm)
Poly(AN-*co*-AA) (P)	22.99	0.241	41.60
TA-Poly(AN-*co*-AA) (FP)	26.31	0.158	47.23

**Table 3:** Adsorption kinetic model constants and correlation coefficients.

Models	Parameters				
PFO	$C_{0}$ (mg/L)	$k_{1}$ (min^−1^)	$q_{e(\exp)}$ (mg/g)	$q_{e(cal)}$ (mg/g)	$R^{2}$
	20	0.0484	3.76	1.32	0.3348
	40	0.0445	7.42	1.45	0.4390
	60	0.0737	11.04	2.99	0.6677
	80	0.0489	14.59	2.54	0.4466
	100	0.0673	18.12	4.86	0.8413
PSO	$C_{0}$ (mg/L)	$k_{2}$ (mg/(g·min))	$q_{e(\exp)}$ (mg/g)	$q_{e(cal)}$ (mg/g)	$R^{2}$
	20	0.0559	3.76	3.89	0.9991
	40	0.0738	7.42	7.51	0.9997
	60	0.0492	11.04	11.22	0.9995
	80	0.0430	14.59	14.75	0.9996
	100	0.0341	18.12	18.36	0.9996
Elovich	$C_{0}$ (mg/L)	β (g/mg)	α (mg/(g·min))		$R^{2}$
	20	1.5032	2.73		0.9502
	40	1.3492	242.33		0.9422
	60	1.1062	3297.24		0.9371
	80	1.1106	106999.96		0.9291
	100	0.9058	165776.93		0.9553
IPD	$C_{0}$ (mg/L)	$k_{IP}$ (mg/(g·min^1/2^))	$C_{IP}$ (mg/g)		$R^{2}$
	20	0.2719	1.45		0.7225
	40	0.3025	4.86		0.7140
	60	0.3467	8.02		0.8357
	80	0.3978	11.06		0.8413
	100	0.4845	13.84		0.8533

**Table 4:** Adsorption isotherm models.

Isotherm	Nonlinear	Linear	Parameters
Langmuir	$q_{e}=\frac{q_{\max}K_{L}C_{e}}{1+K_{L}C_{e}}$	$\frac{C_{e}}{q_{e}}=\frac{1}{K_{L}q_{\max}}+\frac{C_{e}}{q_{\max}}$	$q_{\max}$: monolayer capacity $K_{L}$: constant
Freundlich	$q_{e}=K_{F}C_{e}^{1/n}$	$\ln q_{e}=\ln K_{F}+\frac{1}{n}\ln C_{e}$	$K_{F}$: Freundlich constant n: adsorption intensity

**Table 5:** Adsorption isotherm model constants and correlation coefficients of MB onto TA-poly(AN-*co*-AA).

Model	Langmuir Model			Freundlich Model		
Parameters	$K_{L}$ (L/mg)	$q_{\max}$ (mg/g)	$R^{2}$	$K_{F}$ (L/mg)	n	$R^{2}$
Non-linear	0.0290	440.81	0.9891	15.561	1.253	0.9952
Linear	0.0476	308.64	0.8712	0.028	1.283	0.9993

**Table 6:** Comparison of maximum adsorption capacity of various adsorbent for MB at 298 K.

Adsorbents	$q_{\max}\;(\mathrm{mg}/g)$		References
	Linear	Nonlinear	
Poly(acrylic acid)/polyethersulfone composite	84.82	129.01	[19]
Poly(cyclotriphosphazene-*co*-phloroglucinol)	50.7	-	[58]
Polydopamine microspheres	161.29	-	[59]
Amino group in metal organic frameworks (MIL-53(Al)-NH_2_)	-	188.6	[60]
Poly-melamine-formaldehyde polymer	80.8	-	[61]
Glutaraldehyde-crosslinked poly(vinyl alcohol)/vitamin C-CNTs composite	16.84	18.36	[42]
Dithiocarbamate-functionalized graphene oxide	137	-	[52]
TA-poly(AN-*co*-AA)	308.64	440.82	This work

**Table 7:** Thermodynamic parameters for adsorption of MB onto TA-poly(AN-*co*-AA).

Temperature T (K)	$K_{d}$	Parameters		
		$\Delta G^{o}\;(\mathrm{kJ}/\mathrm{mol})$	$\Delta H^{o}\;(\mathrm{kJ}/\mathrm{mol})$	$\Delta S^{o}(J/\mathrm{mol}\;K)$
298	1.9220	−1.443	−35.588	−114.58
308	1.0793	−0.297	-	-
318	0.6274	0.848	-	-
328	0.5388	1.994	-	-

## Supplementary Materials

The following are available online at https://www.mdpi.com/1996-1944/12/11/1734/s1, Figure S1: IR spectra of a 97:3 poly(AN-*co*-AA) for ODR value.
