# Supplementary material for: Adsorptive Removal of Methylene Blue from Aquatic Environments Using Thiourea-Modified Poly(Acrylonitrile-co-Acrylic Acid)
Source: Materials (Basel). 2019 May 28;12(11):1734. doi: 10.3390/ma12111734 (PMC6600694; doi:10.3390/ma12111734)
Supplement: Supplementary file 1 [file materials-12-01734-s001.pdf]

materials

Article

Adsorptive Removal of Methylene Blue from Aquatic Environments Using Thiourea-Modified Poly(Acrylonitrile-*co*-Acrylic Acid)

Abel Adekanmi Adeyi [1,2], Siti Nurul Ain Md Jamil [3,4,*], Luqman Chuah Abdullah [1,5], Thomas Shean Yaw Choong [1,5], Kia Li Lau [1] and Mohammad Abdullah [6]

1 Department of Chemical and Environmental Engineering, Faculty of Engineering, Universiti Putra Malaysia, UPM Serdang 43400, Malaysia; abeladeyi@abuad.edu.ng (A.A.A.); chuah@upm.edu.my (L.C.A.); csthomas@upm.edu.my (T.S.Y.C.); laukiali@hotmail.com (K.L.L.)
2 Department of Chemical and Petroleum Engineering, College of Engineering, Afe Babalola University Ado-Ekiti, ABUAD, KM. 8.5, Afe Babalola Way, P.M.B. 5454, Ado-Ekiti, Ekiti State, Nigeria
3 Department of Chemistry, Faculty of Science, Universiti Putra Malaysia, UPM Serdang 43400, Malaysia
4 Centre of Foundation Studies for Agricultural Science, Universiti Putra Malaysia, UPM Serdang 43400, Malaysia
5 Institute of Tropical Forestry and Forest Products (INTROP), Universiti Putra Malaysia, UPM Serdang 43400, Malaysia
6 Faculty of Chemical Engineering, Universiti Teknologi Mara, 81750 Masai, Johor darul Takzim, Malaysia; moham3767@johor.uitm.edu.my
* Correspondence: ctnurulain@upm.edu.my; Tel.: +60-123-566-076

Supplementary Data

Calculation of Actual Copolymer Composition

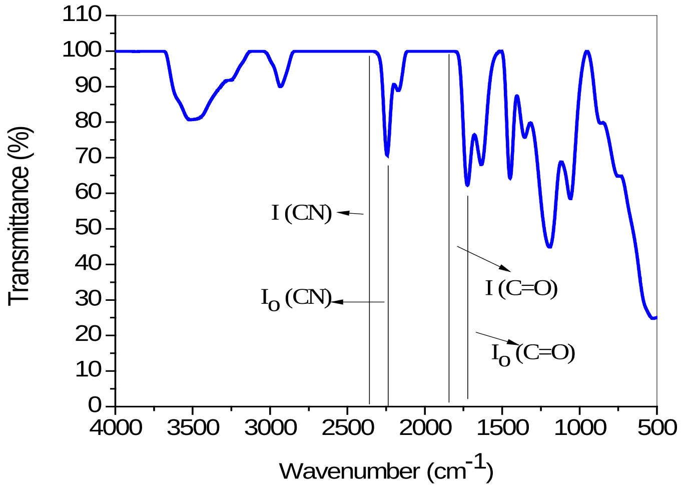

Figure S1. IR spectra of a 97:3 poly(AN-co-AA) for ODR value.

The actual composition of poly(AN-co-AA) copolymer was calculated using optical density ratio (ODR) value to reduce the error of physical variations [1]. The ODR equation is written as

$$ODR = \frac{\left(Absorbance\ ratio\right)_{w(C=O)}}{\left(Absorbance\ ratio\right)_{w(C\equiv N)}} = \frac{\log\left(I_o/I\right)_{w(C=O)}}{\log\left(I_o/I\right)_{w(C\equiv N)}} \quad (S1)$$

Where I and I_o are the intensities of the transmitted and incident radiations, respectively. These are obtained from the infrared (IR) spectrum as shown in Figure S1.

$$ODR = \frac{\log(I_o/I)_{w(C=O)}}{\log(I_o/I)_{w(C\equiv N)}} = \frac{\log(62.24/99.94)_{w(C=O)}}{\log(71.19/99.88)_{w(C\equiv N)}} = 1.399$$

Then, the ODR value was use to substitute the "y" in Equation (S2) [2]

$$x = 16.3y - 8.5y^2 + 2.24y^3 - 1.78 \qquad \text{(S2)}$$

$$x = 10.52 \text{ \% of AA}$$

Thus, the actual composition of AN:AA feed of 97:3 poly(AN-co-AA) was 89.48:10.52

Reference

1. Akbari, S.; Kish, M.H.; Entezami, A.A. Copolymer of acrylonitrile/acrylic acid film dendrigrafted with citric acid: Host/guest properties of dendrigraft/dye complexes in relation to acrylic acid content. *Iran. Polym. J. (English Ed.)* **2011**, *20*, 539–549.
2. Moghadam, S.S.; Bahrami, S.H. Copolymerization of Acrylonitrile-acrylic Acid in DMF-water Mixture. *Iran. Polym. J.* **2005**, *14*, 1032–1041.
